# COVID-19 receptor and malignant cancers: Association of* CTSL* expression with susceptibility to SARS-CoV-2

**DOI:** 10.7150/ijbs.70172

**Published:** 2022-03-06

**Authors:** Lianmei Zhang, Chunli Wei, Dabing Li, Jiayue He, Shuguang Liu, Haoyue Deng, Jingliang Cheng, Jiaman Du, Xiaoyan Liu, Hanchun Chen, Suan Sun, Hong Yu, Junjiang Fu

**Affiliations:** 1 Department of Pathology, Taizhou People's Hospital Affiliated to Nanjing University of Chinese Medicine, Taizhou, Jiangsu Province, China.; 2 Key Laboratory of Epigenetics and Oncology, the Research Center for Preclinical Medicine, Southwest Medical University, Luzhou 646000, Sichuan Province, China.; 3 Department of Pathology, The Affiliated Huaian No. 1 People's Hospital of Nanjing Medical University, Huai'an 223300, Jiangsu Province, China.; 4 Basic Medical School, Southwest Medical University, Luzhou 646000, Sichuan Province, China.; 5 Department of Biochemistry, School of Life Sciences, Central South University, Changsha 410013, Hunan Province, China.

**Keywords:** The *CTSL* gene, Malignant cancers, SARS-Cov-2, COVID-19, Susceptibility, N6, N6-dimethyladenosine (m^6^_2_A), Cordycepin (CD)

## Abstract

CTSL is expressed by cancerous tissues and encodes a lysosomal cysteine proteinase that regulates cancer progression and SARS-CoV-2 entry. Therefore, it is critical to predict the susceptibility of cancer patients for SARS-CoV-2 and evaluate the correlation between disease outcomes and the expression of CTSL in malignant cancer tissues. In the current study, we analyzed CTSL expression, mutation rate, survival and COVID-19 disease outcomes in cancer and normal tissues, using online databases. We also performed immunohistochemistry (IHC) to test CTSL expression and western blot to monitor its regulation by cordycepin (CD), and N6, N6-dimethyladenosine (m^6^_2_A), respectively. We found that *CTSL* is conserved across different species, and highly expressed in both normal and cancer tissues from human, as compared to ACE2 or other proteinases/proteases. Additionally, the expression of CTSL protein was the highest in the lung tissue. We show that the mRNA expression of *CTSL* is 66.4-fold higher in normal lungs and 54.8-fold higher in cancer tissues, as compared to *ACE2* mRNA expression in the respective tissues. Compared to other proteases/proteinases/convertases such as TMPRSS2 and FURIN, the expression of CTSL was higher in both normal lungs and lung cancer samples. All these data indicate that *CTSL* might play an important role in COVID-19 pathogenesis in normal and cancer tissues of the lungs. Additionally, the CTSL-002 isoform containing both the inhibitor_I29 and Peptidase_C1 domains was highly prevalent in all cancers, suggesting its potential role in tumor progression and SARS-CoV-2 entry in multiple types of cancers. Further analysis of the expression of CTSL mutant showed a correlation with FURIN and TMPRSS2, suggesting a potential role of CTSL mutations in modulating SARS-CoV-2 entry in cancers. Moreover, high expression of *CTSL* significantly correlated with a short overall survival (OS) in lung cancer and glioma. Thus, CTSL might play a major role in the susceptibility of lung cancer and glioma patients to SARS-CoV-2 uptake and COVID-19 severity. Furthermore, CD or m^6^_2_A inhibited CTSL expression in the cancer cell lines A549, MDA-MB-231, and/or PC3 in a dose dependent manner. In conclusion, we show that CTSL is highly expressed in normal tissues and increased in most cancers, and CD or m^6^_2_A could inhibit its expression, suggesting the therapeutic potential of targeting CTSL for cancer and COVID-19 treatment.

## Introduction

The *CTSL* (*Cathepsin L*) gene, also known as *MEP*, *CATL*, and *CTSL1*, is located in the chromosome region 9q21.33. The GenBank access number for *CTSL* gene is NM_001912.5 and CTSL protein is NP_001903.1. The *CTSL* gene encodes a 37,564 (Da) lysosomal cysteine proteinase consisting of 333 amino acids, which plays a critical role in intracellular protein catabolism. CTSL belongs to the peptidase C1 family, which forms a disulfide-linked dimer of heavy and light chains 1. As a proteinase, the substrates of CTSL include elastin, collagen, and alpha-1 protease inhibitor and thus it is implicated in several pathologic processes, including myofibril necrosis and tumor progression. Upregulation of CTSL correlates with metastatic aggressiveness and poor prognosis in cancer patients 2. CTSL expression was shown to be elevated in glioblastoma multiforme (GBM) tissue as compared to the normal brain tissue 3. Importantly, CTSL can proteolytically cleave the S1 subunit on severe acute respiratory syndrome coronavirus 2 (SARS-CoV-2) spike protein, which is necessary for viral entry into the host cells 4-6. Middle East Respiratory Syndrome (MERS) and Gingival overgrowth are some of the other diseases associated with CTSL 7-9.

COVID-19 (coronavirus disease 2019) has quickly spread since December 2019 and the number of cases due to SARS-CoV-2 infection have been rising worldwide 10-13. At the end of January 2022, more than 375 million people were diagnosed with SARS-CoV-2 infection and the confirmed deaths were over 5.6 million worldwide (https://coronavirus.jhu.edu/). The critical event promoting the entry of genetic material from SARS-CoV-2 into the host cells is the activation of S-protein by host proteinases/proteases, which enables its binding to the host cell receptors. The cleavage between the residues Thr696 and Met697 in S-protein domains of S1-S2 by CTSL promotes the release of the viral genome into the host cells 14. Previous *in vitro* studies have demonstrated that the inhibition of CTSL decreases the entry of SARS-CoV-2 into the host cells by more than 76%, indicating the importance of CTSL in mediating the viral entry. Therefore, targeting CTSL could be a potential strategy for treating SARS-CoV-2 infection 15-17.

The nucleoside antimetabolite cordycepin (CD), derived from the fungal extracts of* Cordyceps militaris,* has been reported to show broad spectrum anti-virial, anti-cancer, anti-inflammatory, hepato-protective, antidepressant, and neuro-protective activity 18, 19. CD showed strong binding affinities with SARS-CoV-2 S-protein and Mpro proteins 20. CD was also reported to inhibit the expression of FURIN, a SARS-CoV-2 receptor, on cancer cell lines in a dose dependent manner 21. N6,N6-Dimethyladenosine (m^6^_2_A) is a modified ribonucleoside previously found in rRNA and also presented in tRNA from *mycobacterium bovis* Bacille Calmette-Guérin 22.

Regulation of CTSL expression by Chinese medicine has shown its potential role in tumorigenesis, cancer progression and SARS-CoV-2 infection. However, the impact of CTSL expression in SARS-CoV-2 infected cancer patients is still unknown. Thus, it is important to predict the susceptibility of cancer patients for SARS-CoV-2 entry and the disease outcome by accessing the expression of CTSL in different tumor tissues. It is also not known whether CD or m^6^_2_A regulates *CTSL* expression. In the current work, we analyzed the expression profile of *CTSL* in different types of tumor tissues and matched normal tissues, in order to predict its potential role as a therapeutic marker. Moreover, *in vitro* studies showed that CD and m^6^_2_A could suppress CTSL expression.

## Materials and Methods

### Homology analysis

Homologs of CTSL in humans (NP_001903.1 in protein and NM_001912.5 in gene in GenBank, Ensembl ID: Ensembl:ENSG00000135047) and others species were determined from the NCBI (National Center for Biotechnology Information) (https://www.ncbi.nlm.nih.gov/homologene?Db=homologene&Cmd=Retrieve&list_uids=129366) as previously described 23, 24. UniProtKB/Swiss was used to determine CTSL domains (Prot: P07711).

### Online databases for CTSL expressions

The Human Protein Atlas (HPA) was used to predict the *CTSL* gene and protein expressions in the normal and tumor tissues (https://www.proteinatlas.org/ENSG00000135047-CTSL) 25-27. The gene expression of *CTSL* in multiple cancer tissues and matched normal tissues were performed from Genotype-Tissue Expression (GTEx) and The Cancer Genome Atlas (TCGA) databases using GEPIA 2 analysis (Gene Expression Profiling Interactive Analysis) (http://gepia2.cancer-pku.cn/#analysis) 28. GEPIA 2 was also used to compare the gene expressions for *FURIN*, *CTSL* and *TMPRSS2* (*transmembrane protease serine 2*) in cancer tissues and matched normal tissues. For analysis of CTSL isoform usage/distribution and structure of domains from different cancer tissues, GEPIA2 analysis was also conducted in the large datasets of TCGA and GTEx. Survival analysis of CTSL expressions from TCGA database was also obtained from HPA.

### *CTSL* mutation analysis and effect on COVID-19 receptor expression

Gene mutation modules for *CTSL* were performed by TIMER2.0 (http://timer.comp-genomics.org/), and compared to the expression of other receptors, such as *ADAM17 (ADAM Metallopeptidase Domain 17)*, *HSPA5 (Heat Shock Protein Family A (Hsp70) Member 5)*,* ACE2 (Angiotensin Converting Enzyme 2)*, *TMPRSS2*,* FURIN*, and *CTSL*, in order to evaluate the importance of SARS-CoV-2 infection.

### Immunohistochemistry (IHC) assays

The methods for immunohistochemistry (IHC) in formalin-fixed, paraffin-embedded lung and breast cancer tissue sections from Chinese patients were described previously 21, 26, 27, 29. The CTSL antibody (Catalog No. ABIN1172740) for IHC was purchased from antibodies-online GmbH (Aachen, Germany). The color images of the antibody stained tissue sections were obtained using microscopy. The immune positive rate was generally based on the percentage of positive cells and the staining intensity. It was divided into 0, < 5%; 1, 5% ~ 25%; 2, 25% ~ 50%; 3, 50% ~ 75% and 4, > 75%. Immunohistochemical color intensity was divided into: 1, weak; 2, medium; and 3, intense. The two scores were multiplied, and the product was defined as the immunohistochemical score. This evaluation was carried out by two independent pathologists in a blinded fashion.

### Western blotting and semi-quantitative RT-PCR analysis

Cordycepin (CD) was purchased from Must Bio-Technology Co. Ltd in Chengdu, Sichuan, P. R. China. The lung cancer cell line A549, prostate cancer cell line PC3 and triple-negative breast cancer cell line MDA-MB-231 were used in this study. Western blotting for CTSL protein expression was conducted in A549, MDA-MB-231 and PC3 with CD treatments (0, 10µm, 20µm, 40µm) for 24 hours, which were described previously 21. PC3 prostate cancer cells were treated with N6, N6-dimethyladenosine (m^6^_2_A) (0, 10µm, 20µm, 40µm) for 24 hours. The CTSL antibody for western blotting was purchased from Abcam (Catalog No. ab200738). Tubulin (anti-β-Tubulin) antibody (Sigma-Aldrich, catalog No. T0198) was used as the internal control. Western blotting was described previously 29-31. The semi-quantitative RT-PCR analysis was also performed using the above treated cells. Primers for RT-PCR were as follows: RT-CTSL-L, 5'-agggaagggaaacacagctt-3', RT-CTSL-R, 5'-aagcccaacaagaaccacac-3'. The amplified size was 223bp. *GAPDH* was used as an internal control. All experiments were repeated three times.

### Cycloheximide (CHX) chase assays

MDA-MB-231 cell line was used for cycloheximide (CHX, Catalog No. A49960, ACMEC in Shanghai, China) treatment, for the indicated time with or without CD treatment (CD treated for 1 hour at first). Following this, western blotting for CTSL was performed as described above. Tubulin antibody was also used as an internal control. The intensities for CTSL and tubulin bands were quantified by densitometry and analyzed using adobe photoshop CS3 software 32. The experiments were repeated three times.

## Results

### Conservation of CTSL across species

**Figure 1:**
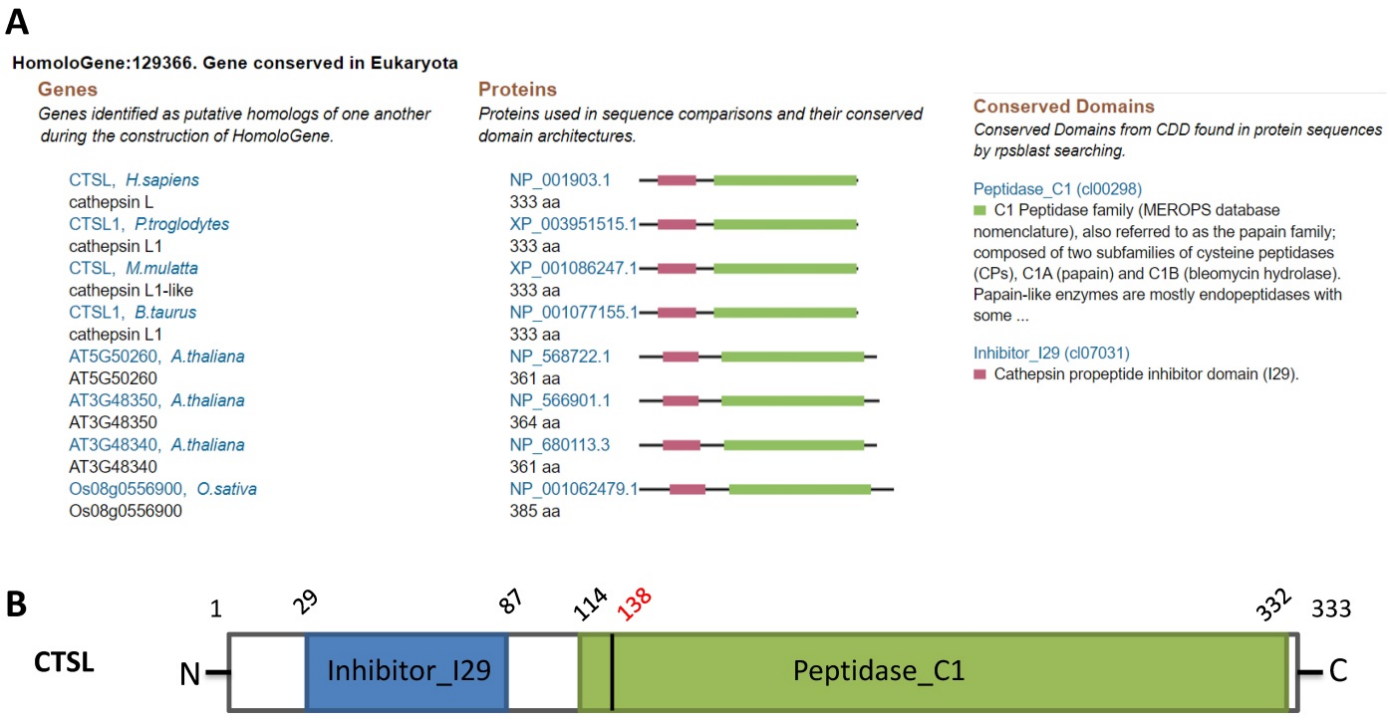
** Homologs and conservation of the CTSL protein. A.** CTSL expression from different species. **B.** Conserved domains of CTSL.

Homology analysis revealed the conservation of CTSL protein across different species such as chimpanzee, Rhesus monkey, cow, A. thaliana, and rice, suggesting a potential role of CTSL in SARS-CoV-2 entry in multiple species (Figure 1A). Structural analysis of CTSL showed two conserved domains, including Inhibitor_I29 (Cathepsin propeptide inhibitor domain (I29)) and Peptidase_C1 (Papain family cysteine protease) (Figure 1B).

### CTSL expression varies across different “normal” tissues

**Figure 2:**
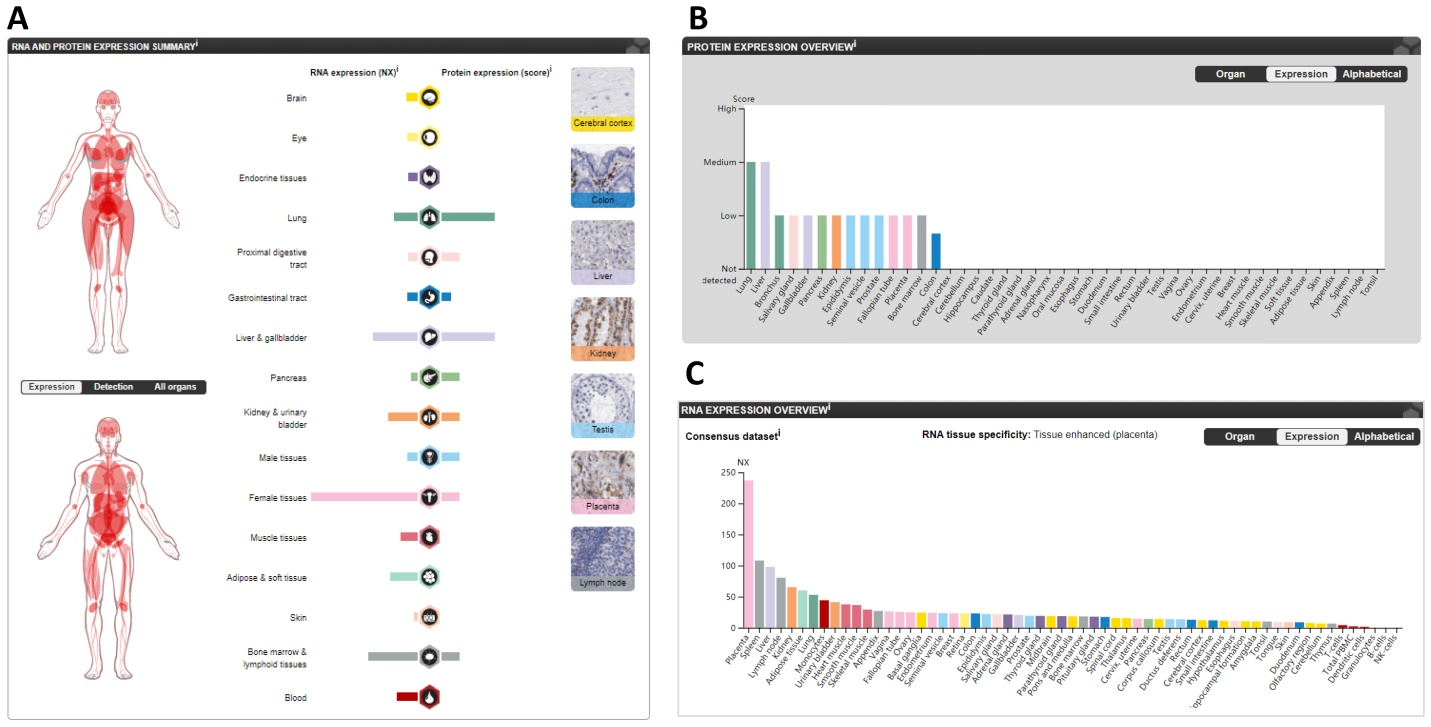
** The expression of CTSL in normal tissues in humans. A.** The summary of CTSL expression in organs. **B.** The CTSL protein expression in normal tissues. **C.** The *CTSL* mRNA expression in normal tissues. NX, consensus normalized expression.

The protein and mRNA expression profiles of CTSL in different tissues from the HPA are summarized in Figure 2A. CTSL protein levels, based on a high, medium, low, or not detected score, revealed two tissues (lungs and livers) with medium expression; eleven tissues with low expression; one tissue (colon) with lower expression; and thirty-one tissues showed no detectable expression (Figure 2B). For mRNA levels, the consensus dataset consisted of normalized expression (NX) levels in different tissue types by combining the transcriptomic datasets of the HPA and GTEx using the internal normalization pipeline. *CTSL* mRNA levels from these consensus datasets revealed a high expression in the placenta with 237.0 NX, followed by the spleen (108.1 NX), and the liver (98.0 NX); noticeably, the lungs had the seventh highest expression (53.1 NX). B-cells and NK-cells were found to have the lowest *CTSL* mRNA expression (Figure 2C). Therefore, our findings show that the expression levels of CTSL varies across different human tissues. High expression of CTSL in the lungs indicates its importance for viral entry into the lungs.

### CTSL isoform usage and structure in cancer tissues

**Figure 3:**
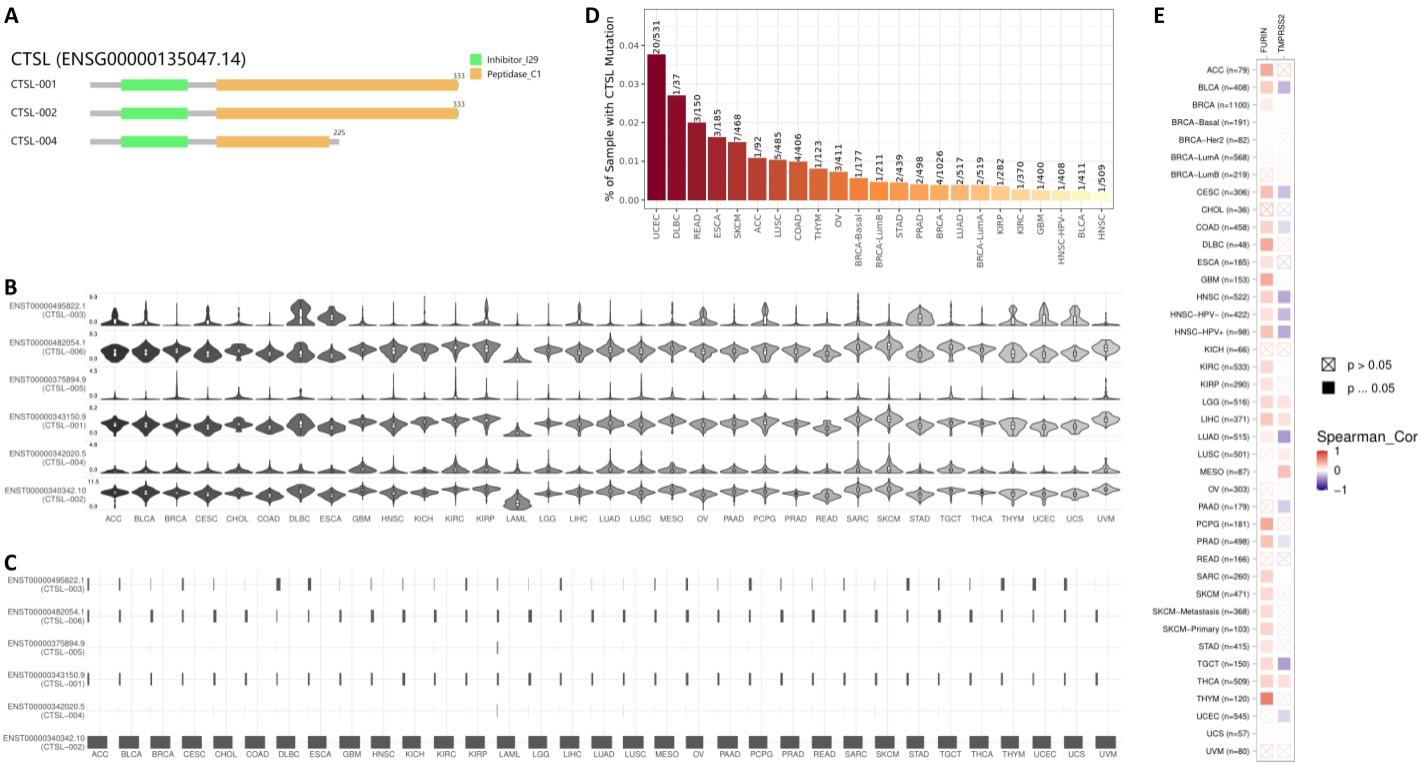
** The usage and the structure of CTSL isoforms in different types of cancers. A.** Structure of CTSL isoforms is shown. Three isoforms and two visualized domains are shown in an interactive plot. Note: information for the following 3 isoforms are missing: ENST00000375894.9, ENST00000482054.1 and ENST00000495822.1. **B & C.** Usage of different CTSL isoforms. The profiles for the distribution of CTSL expression are shown with violin plot in panel B, and isoform usage is shown with bar plot in panel C. The X axis presents isoforms, whereas the Y axis presents the respective cancer types. **D.** Mutation status for* CTSL* in different tumors from TCGA. **E.** The heat maps show the log2 fold expression changes of *FURIN* and *TMPRSS2* in each tumor type. The full names of cancers are shown in right panel of Figure 6.

Expression of different ACE2 isoforms in the airway epithelium contributes differentially to viral susceptibility 33; isoforms for other SARS-CoV-2 receptors may also play similar roles. GEPIA2 database in 33 types of tumor tissues showed three isoforms (Figure 3A). The isoforms CTSL-001 and CTSL-002 contain both the inhibitor_I29 and Peptidase_C1 domains, which is same as shown in Figure 1B. But isoform CTSL-004 only contains the inhibitor_I29 and half of the Peptidase_C1 domain. Information for the other three isoforms ENST00000375894.9 (CTSL-005), ENST00000482054.1 (CTSL-006), and ENST00000495822.1 (CTSL-003) were missing. As for CTSL isoform prevalence, we noticed that all six isoforms were expressed, but at different levels, with CTSL-006 and CTSL-001 being the highest, followed by CTSL-002; and CTSL-006 was the lowest in different tumors (Figure 3B). The usage of isoform CTSL-002 was very high in all cancers; the other five isoforms were found to be minimally expressed (Figure 3C). Based on the distribution of CTSL isoform expression and usage, we concluded that CTSL-002 might play a major role in tumor progression and SARS-CoV-2 entry in different kinds of tumor tissues.

*CTSL* mutation analysis in different tumors showed the highest mutation rate in UCEC (Uterine Corpus Endometrial Carcinoma) (20/531=3.77%), followed by DLBC (Lymphoid Neoplasm Diffuse Large B-cell Lymphoma) (1/37=2.70%), and HNSC (Head and Neck squamous cell carcinoma) showed the lowest (1/509=0.19%) mutation rate (Figure 3D). Further analysis of the correlation of mutant CTSL expression with other proteases/proteinases or convertases such as FURIN and TMPRSS2 were performed. This analysis is mainly based on the log2 fold expression changes of interesting genes in each cancer type. We report that the mutant CTSL expression correlates with elevated FURIN expression in multiple cancers, while there is no such correlation in a few cancers (Figure 3E, FURIN panel); but TMPRSS2 showed either increase or decrease in a few cancers (Figure 3E, TMPRSS2 panel). Considering TMPRSS2 is mainly expressed in prostate cancer, we conclude that CTSL mutations primarily correlated with FURIN expression, which might further regulate SARS-CoV-2 entry in cancers.

### The subcellular localization of CTSL in cells

To determine the subcellular spatial distribution of CTSL in human cells, immunofluorescence (IF) staining was analyzed. The IF staining showed CTSL localization at the Golgi apparatus and vesicles of sparkle signals (Supplementary Figure 1). This subcellular localization at Golgi apparatus and vesicles implies the possible role of CTSL in the cleavage of S-protein, enabling its binding to the host receptors.

### CTSL expression in lung cancer and breast cancer

**Figure 4:**
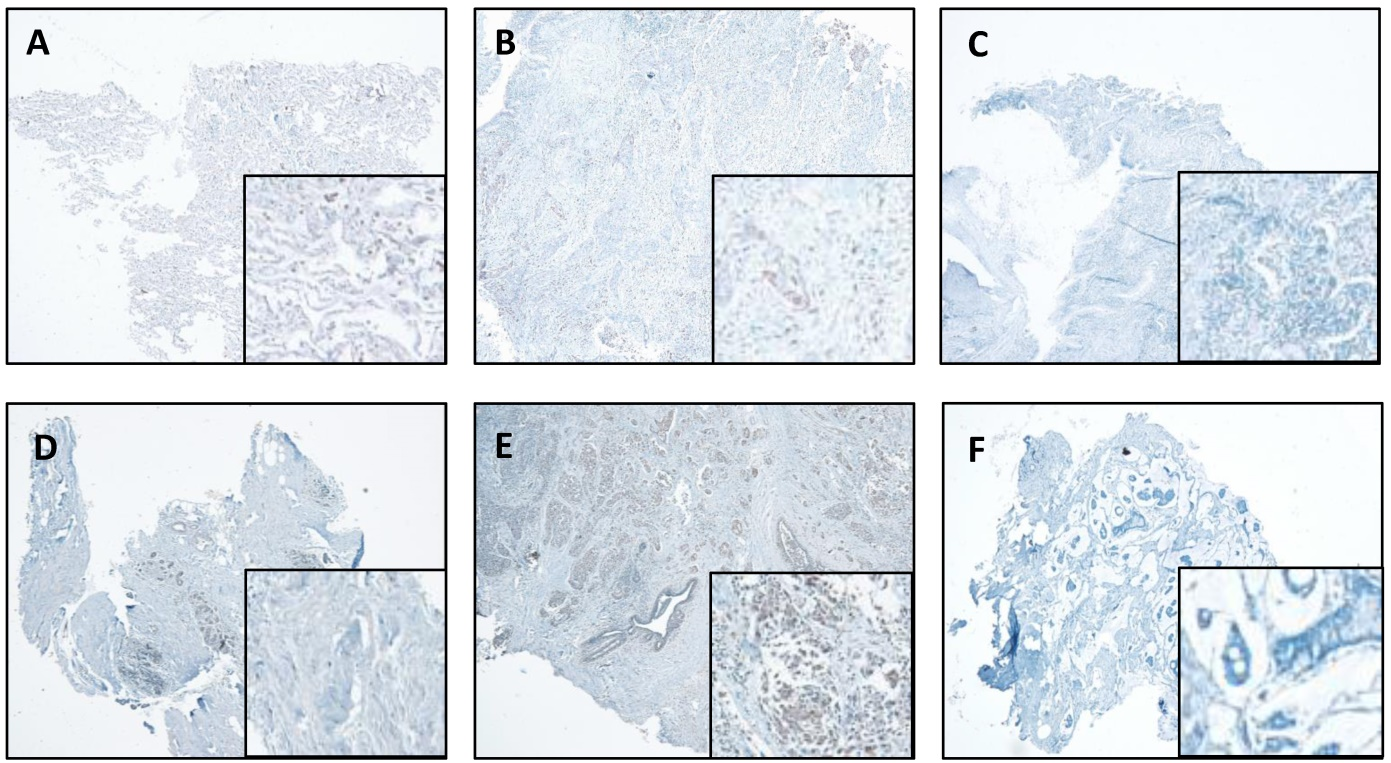
** CTSL expression in normal and tumor tissues of the lung and breast. A.** Representative staining for normal lung tissue from a lung cancer patient. **B.** Representative staining for cancer tissue from a lung cancer patient. **C.** No antibody control sample for normal lung tissue. **D.** Representative staining for normal breast tissues in a breast cancer patient. **E.** Representative staining for cancer tissue from a breast cancer patient. **F.** No antibody control sample for breast cancer tissue. 40X. Enlarged images are presented in the right corners of A~F, respectively. Note that the expression levels are based on the intensity of the staining and the percentage of positive cells.

We also performed IHC in the lung and breast tumors and matched normal tissues. The representative results are shown in Figure 4. We report that IHC staining in the cytoplasm shows moderate intensity in both the normal lung (Figure 4A) and breast tissues (Figure 4D), whereas, slight increase in lung cancer tissues (Figure 4B) and significant increase in breast cancer tissues (Figure 4E) were observed. However, in the absence of antibody, there was no staining detected in the lung and breast cancer tissues (Figure 4C & F).

### Pan-cancer expression of *CTSL* in malignant cancer tissues and matched normal samples

**Figure 5:**
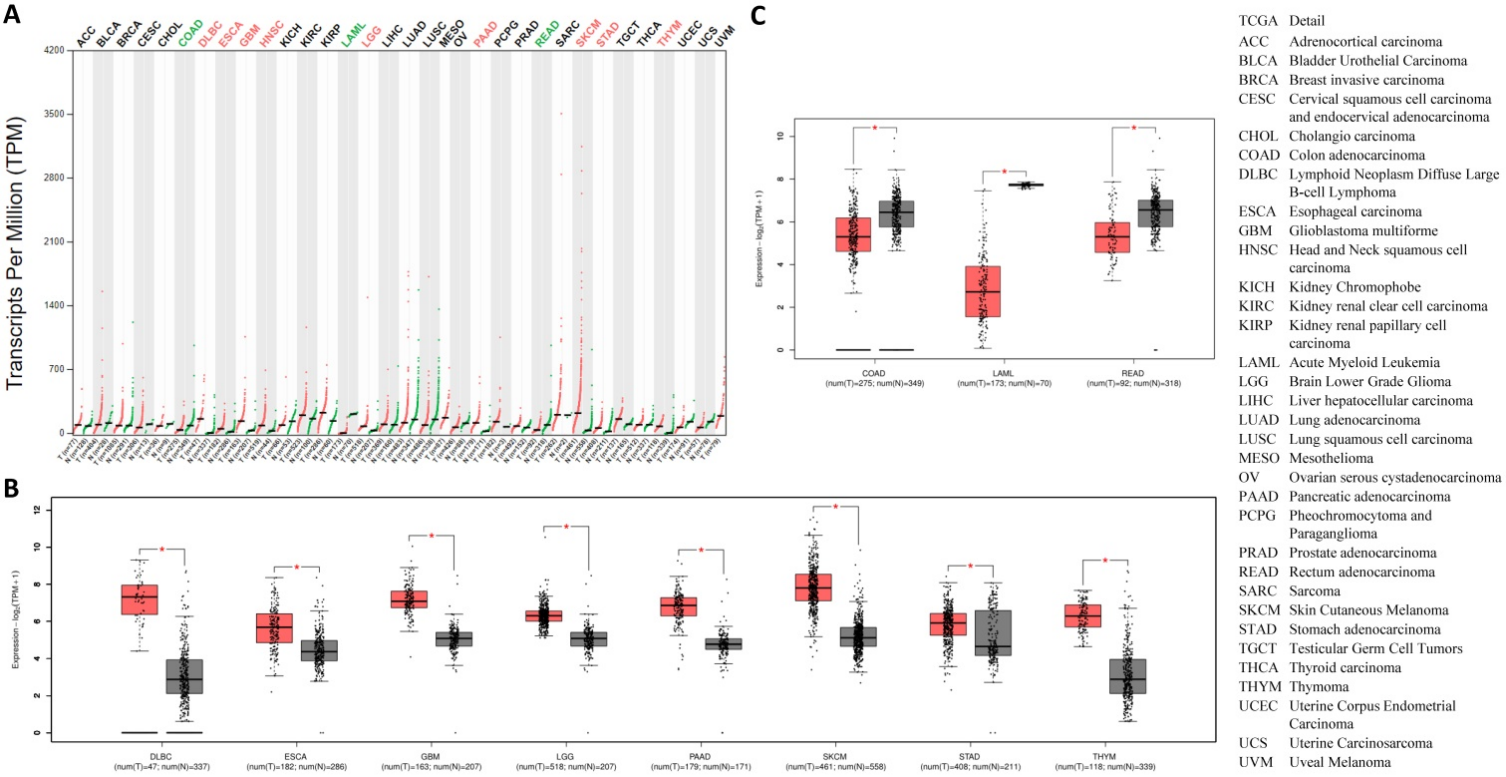
** The expression of *CTSL* in tumor tissues and the corresponding normal tissues. A.** *CTSL* expressions in 33 types of cancer tissues (red files) and the corresponding normal tissues (green files). Green in names indicates decreased expression whereas red in names indicates decreased expression in cancer tissues compared to the corresponding normal tissues. **B.** Expression profiles for *CTSL* in eight tumor tissues and their corresponding normal tissues. **C.** Expression profiles for *CTSL* in three tumor tissues and their corresponding normal tissues (*: p<0.01). The cancer tissue is in red, the normal tissue is in grey. Tissue-wise expressions are used as box plots. Right panel shows the cancerous full names.

Going forward, we quantitatively compared *CTSL* mRNA expression profiles from thirty-three types of cancers and their matched normal tissues, including those of breast and lung. The results revealed that, all types of cancer tissues showed *CTSL* expression; the highest levels were found in SKCM (Skin Cutaneous Melanoma) (Figure 5A). Although most tumor tissues showed an elevated expression of *CTSL* mRNA (Figure 5A), it was significantly elevated in eight tumor types, including DLBC, ESCA (Esophageal carcinoma), LGG (Brain Lower Grade Glioma), GBM (Glioblastoma multiforme), PAAD (Pancreatic adenocarcinoma), SKCM, STAD (Stomach adenocarcinoma) and THYM (Thymoma) (Figure 5A in red, 5B, p<0.01). On the other hand, *CTSL* mRNA levels were dramatically decreased in three other cancer types, including COAD (Colon adenocarcinoma), LAML (Acute myeloid leukemia) and READ (Rectum adenocarcinoma) (Figure 5A in green, 5C, p<0.01). In conclusion, our findings suggest that CTSL may play an important role in the uptake of SARS-CoV-2 in most of the tumor tissues.

### Comparison of the expression levels of *ADAM17*, *HSPA5*, *ACE2*, *TMPRSS2*, *FURIN* and *CTSL* in tumor and normal tissues

**Figure 6:**
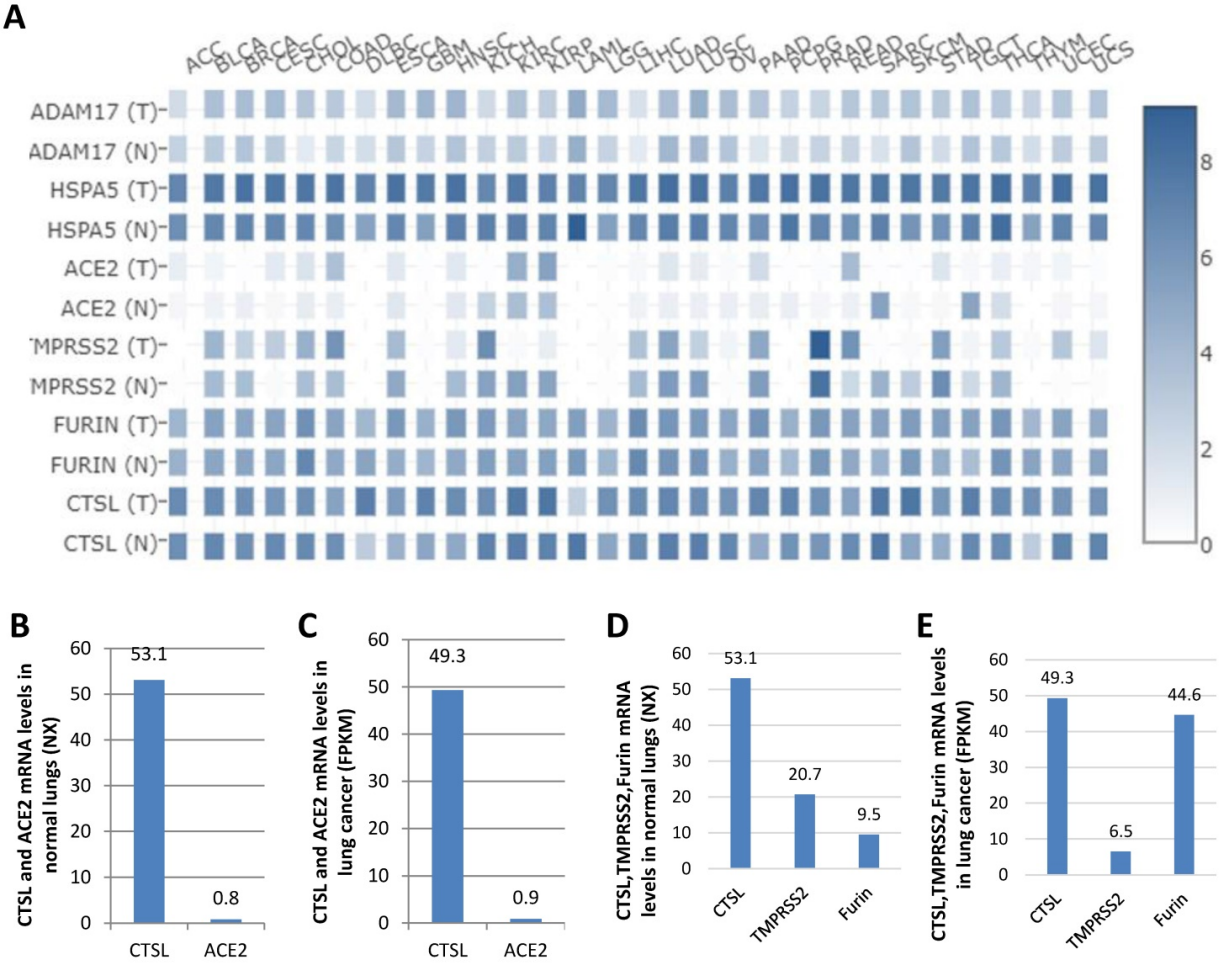
** Comparison of the mRNA expression levels of *ADAM17, HSPA5, ACE2, TMPRSS2, FURIN* and* CTSL* in thirty-one cancers and their matched normal tissues. A.** Comparison of mRNA expression of *ADAM17, HSPA5, ACE2, TMPRSS2, FURIN* and* CTSL* in 31 tumors and their matched normal tissues. “T” represents cancer tissues and “N” represents normal tissues. **B.** Comparison of mRNA expression of between *ACE2* and *CTSL* in normal tissues of the lungs. **C.** Comparison of mRNA expression between* ACE2* and* CTSL* in cancer tissues of the lungs. **D.** Comparison of mRNA expression between *TMPRSS2, FURIN* and *CTSL* in normal tissues of the lungs. **E.** Comparison of mRNA expression between* TMPRSS2, FURIN* and* CTSL* in cancer tissues of the lungs. The consensus dataset consists of normalized expression (NX) levels from different tissue types by combining the transcriptomic datasets of HPA and GTEx, using the internal normalization pipeline, or relative expression levels.

ADAM17, HSPA5, ACE2, TMPRSS2, and FURIN are all viral receptors, which are essential for SARS-CoV-2 uptake 23, 28, 34. The expression levels of these genes were analyzed in different tumor tissues from TCGA datasets. We found that mRNA expression of *HSPA5* was the highest, followed by *FURIN* and *CTSL* in the majority of the tumor tissues, and *ACE2* expression was the lowest (Figure 6A), demonstrating that CTSL might facilitate tumorigenesis and SARS-Cov-2 entry in most cancers.

ACE2 was reported as the most critical functional receptor for SARS-CoV-2 entry into the lungs 35, 36. Thus, we compared the expression of *CTSL* and *ACE2* mRNA in normal lungs and lung cancers. The results showed that *ACE2* expression was approximately 0.8 NX and 0.9 FPKM in normal lungs and lung cancers, respectively. However, *CTSL* expression value was 53.1 NX and 49.3 FPKM, in the respective tissues, which was 66.4-fold (53.1/0.8=66.4) higher than *ACE2* mRNA expression in normal lungs (Figure 6B), and 54.8-fold (49.3/0.9=54.8) higher than *ACE2* mRNA expression in the lung cancers (Figure 6C). CTSL, TMPRSS2 and FURIN belong to proteinase/protease family. We further compared the expression levels of *CTSL*, *TMPRSS2*, and *FURIN* in normal lungs and lung cancers. We found the expression of *CTSL* to be the highest in both tissues (Figure 6C & D). In conclusion, these data demonstrate that *CTSL* may play an important role in COVID-19 pathogenesis in normal and cancerous lung tissues.

### Prognostic value of *CTSL* expression for overall survival in cancer patients

**Figure 7:**
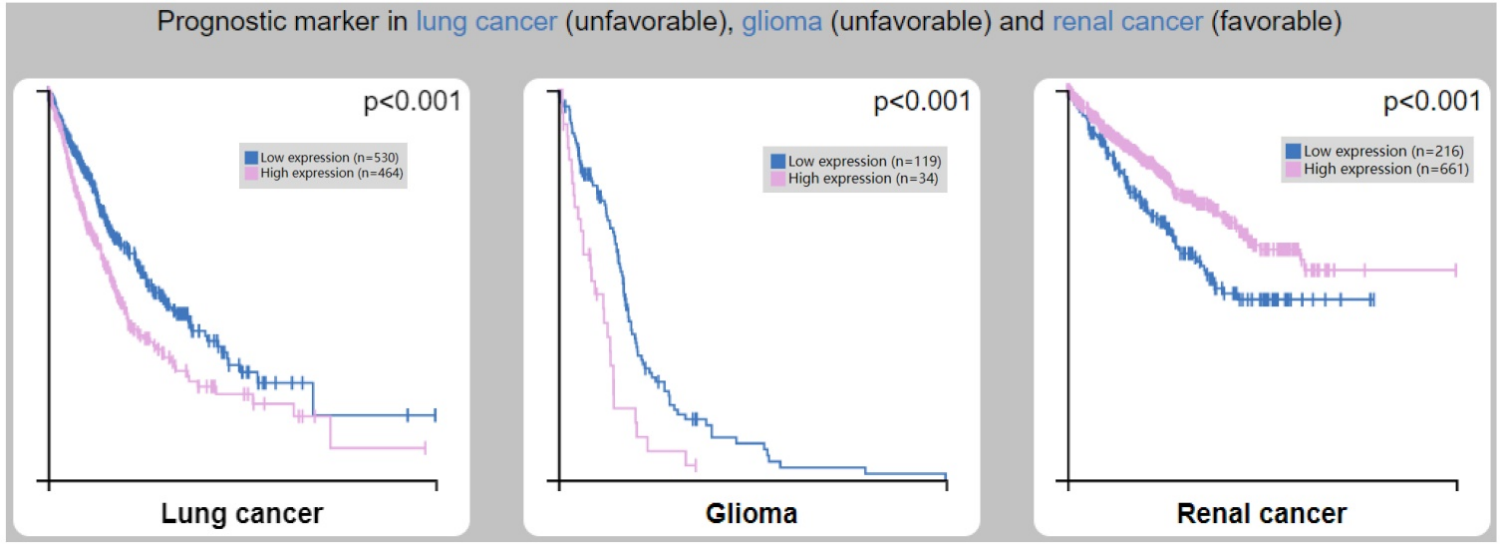
Correlation between CTSL expression and overall survival (OS) in patients with lung cancer (left panel), glioma (middle panel) and renal cancer (right panel). Note: Based on the FPKM (Fragments Per Kilobase Million) value of the *CTSL* gene, cancer patients were classified into two groups (high and low expression) and the correlations between expression level and patient survival were evaluated.

We analyzed the clinical correlation between *CTSL* expression and overall survival (OS) outcomes in HPA (Figure 7). We found that a high expression of *CTSL* was significantly correlated with a short OS in lung cancer (Figure 7A, p<0.001) and glioma patients (Figure 7B, p<0.001). On the other hand, high CTSL expression significantly correlated with a long OS in renal cancer patients (Figure 7C, p<0.001). Thus, CTSL expression may be an unfavorable prognostic marker for survival in lung cancer and glioma patients, and a favorable prognostic marker for survival in renal cancer patients.

### Regulation of CTSL protein expression by Cordycepin (CD) or N6, N6-dimethyladenosine (m^6^_2_A) in cancer cell lines

**Figure 8:**
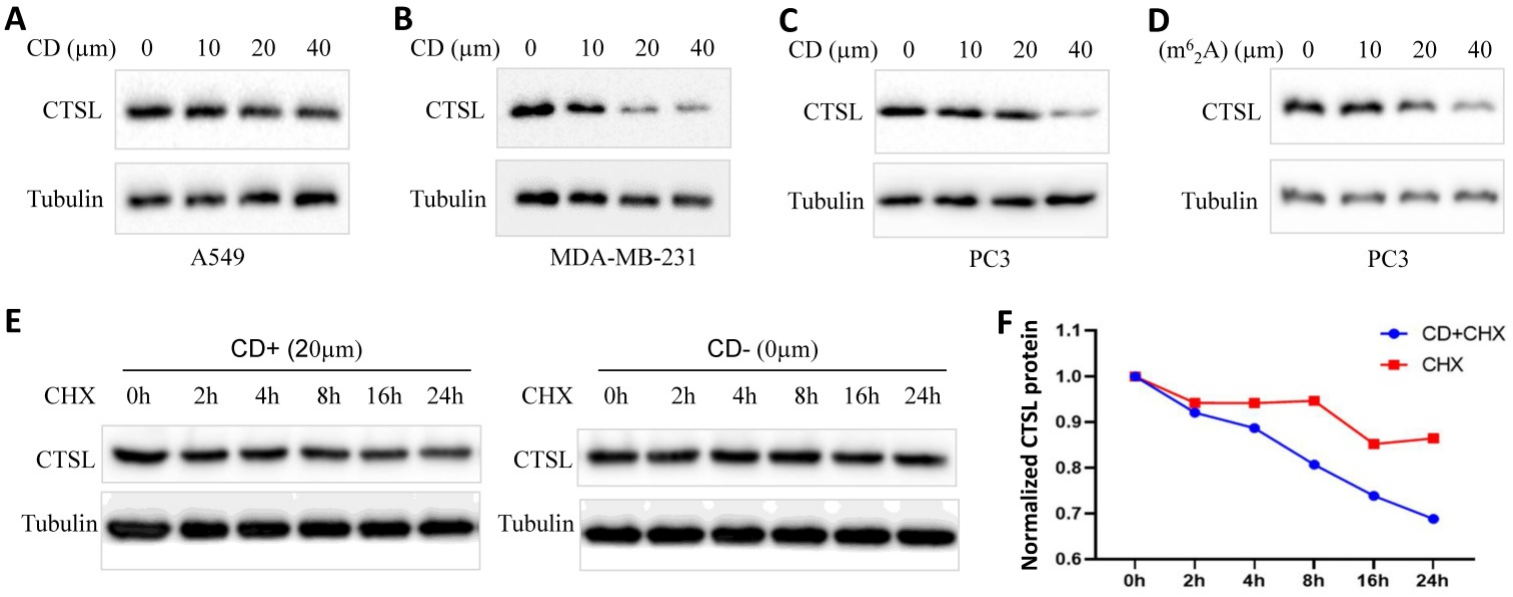
** CD or m^6^_2_A suppresses CTSL expression in cancer cell lines. A.** CTSL protein levels after CD treatment in A549 lung cancer cell line. **B.** CTSL protein levels after CD treatment in MDA-MB-231 triple-negative breast cancer cell line. **C.** CTSL protein levels after CD treatment in PC3 prostate cancer cell line. **D.** CTSL protein levels after N6, N6-dimethyladenosine (m^6^_2_A) treatment in prostate cancer cell line PC3. **E.** CTSL protein stability after CHX treatment with or without CD treatment. Left panel shows CD treatment, right panel shows without CD treatment. **F.** The quantitative results from E. Red line shows CHX treatment only, blue line shows CHX treatment together with CD treatment. The final concentration for CHX treatments were 40 µg/ml.

To explore the possibility of using CD or m^6^_2_A as therapeutics against SARS-CoV-2, we analyzed the change in the expression of CTSL protein in cancer cell lines treated with these agents (Figure 8). We found that CD inhibited CTSL protein levels in a dose dependent manner in the lung cancer cell line A549, triple-negative breast cancer cell line MDA-MB-231, and prostate cancer cell line PC3, respectively (Figure 8A-C). However, treatment with CD did not cause any change in *CTSL* mRNA levels in the same cell lines (Supplementary Figure 2 and data not shown). m^6^_2_A inhibited CTSL protein levels in a dose dependent manner in the prostate cancer cell line PC3 (Figure 8D). These data indicate that CD inhibits CTSL protein levels at the translational level, potentially by degrading CTSL protein. Following this, we performed chase assays with CHX treatment, with and without CD treatments, in MDA-MB-231 cell line. The results showed that, CD treatment reduced the stability of CTSL protein (Figure 8E & F), suggesting that CD degraded CTSL protein. In conclusion, these results suggest that both CD and m^6^_2_A might have a therapeutic potential as anti-SARS-CoV-2 agents through the suppression of CTSL protein expression.

## Discussion

Cancer patients are more susceptible to SARS-CoV-2 infection than those without cancer, and consequently are more likely to become severely ill or die when suffering from this viral infection 37-40. Previous systematic reviews have reported an increased fatality in COVID-19 patients with cancer than those without cancer 39, 41. Given this discrepancy, we sought to evaluate the expression levels of viral entry receptors in various cancer tissues, as cancerous pathology might affect COVID-19 susceptibility and illness 42, 43.

In this study, we found *CTSL* to be highly conserved across different species. We also found it to be highly expressed in normal and cancer tissues, especially in comparison to ACE2, an important receptor for SARS-Cov-2, as well as in comparison to other proteases. CTSL expression was found to be the highest in the lungs. We further compared the expression of *CTSL* and *ACE2* mRNA in normal lungs and lung cancers and found that *CTSL* expression was 66.4-fold higher in normal lungs and 54.8-fold higher in lung cancer tissues, as compared to *ACE2* mRNA levels in the respective tissues. By further comparison of CTSL, TMPRSS2, and FURIN, all of which are proteinase/protease/convertases 43, 44, we found the expression of CTSL to be the highest in both in normal lungs and lung cancers. All these data indicate a potential role of *CTSL* in COVID-19 pathogenesis in normal and cancerous tissues of the lungs. Furthermore, the CTSL-002 isoform containing both inhibitor_I29 and Peptidase_C1 domains is highly expressed in all cancers, suggesting its potential role in tumor progression and SARS-CoV-2 entry. We further analyzed the correlation between CTSL mutations and the expression of other proteases/proteinases/convertases such as FURIN and TMPRSS2, and found a positive correlation between CTSL and FURIN expression in multiple cancers. Thus CTSL mutations may further regulate SARS-CoV-2 entry in cancer tissue. Of note, we did not find any promoter methylation modification in the pan-cancer analysis. In the clinical setting, we found that high expression of *CTSL* was significantly correlated with a short OS in lung cancer and glioma patients. Thus CTSL may play a vital role in the susceptibility to SARS-CoV-2 entry and severity of COVID-19 clinical symptoms, particularly in lung cancer and in glioma patients.

CTSL is known to play a role in cancer invasion and metastasis, inflammation, renal disease, diabetes, bone diseases, atherosclerosis, viral infection, as well as other diseases. CTSL inhibitors or chloroquine has been shown to significantly reduce the viral replication. Therefore, CTSL could be a therapeutic target for both cancers and COVID-19 15, 45. *In vitro* expression analysis showed that CD or m^6^_2_A suppressed CTSL protein expression in a dose dependent manner. This is the first study identifying the inhibition of CTSL by nucleoside derivatives, CD and m^6^_2_A, suggesting their role as anti-SARS-CoV-2 agents through CTSL inhibition.

## Conclusions

CTSL expression was high in normal tissues and was increased in multiple cancer types. Both CD and m^6^_2_A suppressed its expression, implying their therapeutic potential in preventing SARS-CoV-2 invasion and cancer progression. Our study highlights the value of targeting CTSL as a therapeutic strategy to combat cancer and COVID-19 pandemic.

## Supplementary Material

Supplementary figures.
