# Supplementary material for: COVID-19 receptor and malignant cancers: Association of CTSL expression with susceptibility to SARS-CoV-2
Source: Int J Biol Sci. 2022 Mar 6;18(6):2362–71. doi: 10.7150/ijbs.70172 (PMC8990473; doi:10.7150/ijbs.70172)
Supplement: Supplementary file 1 — Supplementary figures. [file ijbsv18p2362s1.pdf]

Supplementary figures

Supplementary figure 1

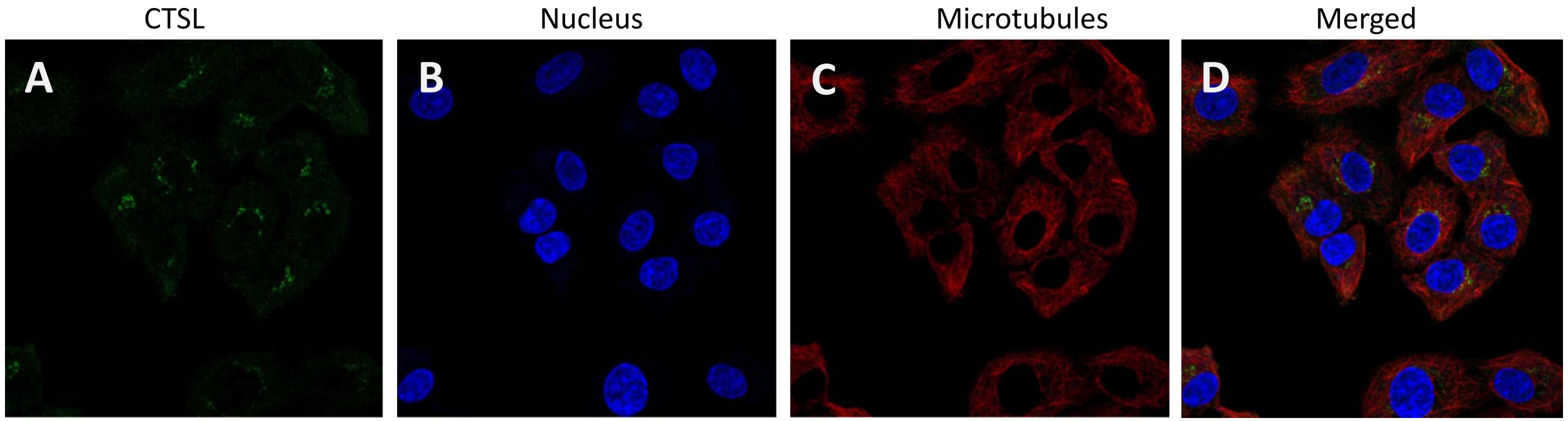

Supplementary figure 1. The CTSL cellular localization in the human cell line A549 (https://www.proteinatlas.org/ENSG00000135047-CTSL/subcellular#human). A. CTSL immunofluorescent staining. B. Nucleus staining. C. Microtubule staining. D. Merged from A&B&C.

Supplementary figure 2

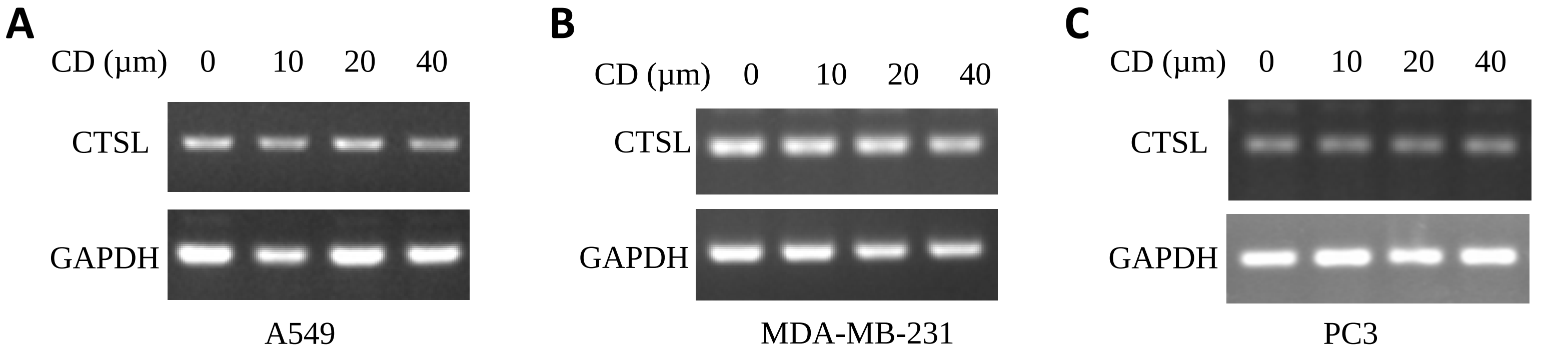

Supplementary figure 2. CD suppresses *CTSL* mRNA expression in cancer cell lines. A. The mRNA levels in lung cancer cell line A549. B. The mRNA levels in triple-negative breast cancer cell line MDA-MB-231.
